# Revelando o Gigante Oculto: Um Caso de Ectasia da Artéria Coronária Direita e Fístula para o Seio Coronário numa Doente Assintomática

**DOI:** 10.36660/abc.20240574

**Published:** 2025-07-01

**Authors:** Inês Ferreira Neves, André Ferreira, Inês Almeida, Tânia Branco Mano, Lídia de Sousa

**Affiliations:** 1 Hospital de Santa Marta Lisboa Portugal Hospital de Santa Marta, Lisboa – Portugal

**Keywords:** Cardiopatias Congênitas, Fístula Arteriovenosa, Seio Coronário, Vasos Coronários

Uma mulher de 62 anos, assintomática e sem histórico médico significativo, foi encaminhada para um ecocardiograma transtorácico (ETT) de rotina. O ETT não apresentou achados anormais, mas mostrou, em vista subcostal, três imagens redondas adjacentes à parede lateral do átrio direito. O Doppler colorido mostrou que estas estavam aparentemente vascularizadas (Figura 1, painéis A e B, vídeos suplementares 1 e 2). Um ecocardiograma transesofágico mostrou uma artéria coronária direita (ACD) gigante, aparentemente originária do óstio da ACD, com fluxo turbulento em seu interior (Figura 1, painel C, vídeo suplementar 3). Não foi possível observar shunts ou fístulas nesta modalidade de imagem. Diante da suspeita de fístula para a ACD, foi realizada angiotomografia coronária, que demonstrou ACD ectásica, de origem normal, diâmetro de 10,5 mm e trajetória tortuosa, com fistulação para o seio coronário em seu segmento final (Figura 1 painéis D a F). O cateterismo cardíaco direito demonstrou shunt esquerda-direita não significativo (relação Qp: Qs de 1,60). A ressonância magnética cardíaca não apresentou evidências de defeitos de perfusão durante a hiperemia, excluindo assim o "fenômeno do roubo" coronário. Após discussão em equipe multidisciplinar, como o paciente era assintomático e a fístula não apresentava significância hemodinâmica, nenhum tratamento invasivo foi realizado neste momento.

**Figura 1:**
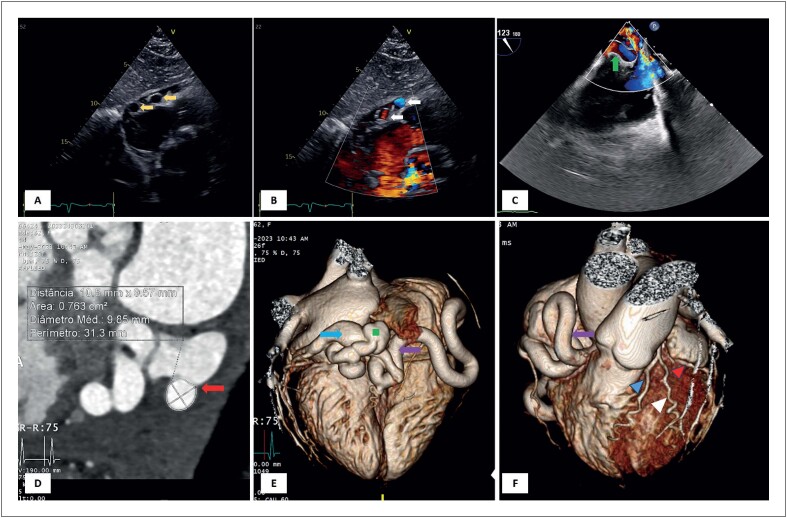
Ectasia da Artéria Coronária Direita e Fístula para o Seio Coronário. Painel A: Ecocardiograma transtorácico (ETT) em vista subcostal, mostrando estruturas pericárdicas redondas (setas amarelas). Painel B: ETT com Doppler, mostrando vascularização das estruturas (setas brancas). Painel C: Ecocardiograma transesofágico mostrando uma artéria coronária direita gigante (ACD) (seta verde) com fluxo turbulento em seu interior. Painel D: Angiografia coronária por tomografia computadorizada (ATC) mostrando uma ACD ectásica com fistulação para o seio coronário (SC) (seta vermelha). Painel E: Vista posterior da ATC mostrando o SC (seta azul), a ACD ectásica (seta roxa) e a localização da fístula (quadrado verde). Painel F: Vista anterior da ATC mostrando a ACD (seta roxa), a artéria descendente anterior (ponta de seta azul), uma artéria marginal (ponta de seta branca) e a artéria circunflexa (ponta de seta vermelha).

**Vídeo 1:** 

As fístulas arteriovenosas coronárias são raras, com incidência de 0,002%.^1,2^ A maioria delas é congênita^2^ e assintomática na apresentação.^3^ O roubo coronário pode levar a várias complicações, incluindo isquemia.^1,3^ A abordagem do tratamento permanece controversa. A presença de sintomas, shunt esquerda-direita significativo e fístula grande são as indicações mais frequentes para abordagem percutânea ou cirúrgica.^4,5^

## Data Availability

Os conteúdos subjacentes ao texto da pesquisa estão contidos no manuscrito.
