# Supplementary material for: Revelando o Gigante Oculto: Um Caso de Ectasia da Artéria Coronária Direita e Fístula para o Seio Coronário numa Doente Assintomática
Source: Arq Bras Cardiol. 2025 Jul 1;122(6):e20240574. [Article in Portuguese] doi: 10.36660/abc.20240574 (PMC12269894; doi:10.36660/abc.20240574)
Supplement: Translation

# Translation: Silent Giant: Right Coronary Artery Ectasia with a Hidden Fistula to the Coronary Sinus in an Asymptomatic Patient

**DOI:** 10.36660/abc.20240574i

**Authors:** Inês Ferreira Neves, André Ferreira, Inês Almeida, Tânia Branco Mano, Lídia de Sousa

**Affiliations:** 1 Lisboa Portugal Hospital de Santa Marta, Lisboa – Portugal

**Keywords:** Congenital Heart Defects, Arteriovenous Fistula, Coronary Sinus, Coronary Vessels

A 62-year-old woman, asymptomatic and without significant medical history, was referred to a routine transthoracic echocardiogram (TTE). The TTE had no abnormal findings but showed, in subcostal view, three round images adjacent to the lateral wall of the right atrium. Color Doppler showed that these were apparently vascularized (Figure 1, panels A and B, supplemental videos 1 and 2). A transesophageal echocardiogram showed a giant right coronary artery (RCA), apparently originating from the RCA ostium, with turbulent flow inside (Figure 1, panel C, supplemental video 3). It was not possible to observe shunts or fistulae in this imaging modality. Given the suspicion of a fistula to the RCA, computed tomography coronary angiography was performed, showing an ectatic RCA with a normal origin, a diameter of 10,5 mm, and a tortuous trajectory, with fistulation to the coronary sinus in its final segment (Figure 1 panels D to F). Right heart catheterization demonstrated a non-significant left-to-right shunt (Qp: Qs ratio of 1.60). Cardiac magnetic resonance imaging (MRI) had no evidence of perfusion defects during hyperemia, thus excluding the coronary "steal phenomenon". After a multidisciplinary team discussion, since the patient was asymptomatic and the fistula had no hemodynamic significance, no invasive treatment was performed at this time.

**Figure 1:** Right Coronary Artery Ectasia and Fistula to the Coronary Sinus. Panel A: Transthoracic echocardiogram (TTE) in subcostal view, showing round pericardial structures (yellow arrows). Panel B: TTE with Doppler, showing vascularization of the structures (white arrows). Panel C: Transesophageal echocardiogram showing a giant right coronary artery (RCA) (green arrow) with turbulent flow inside. Panel D: Computed tomography coronary angiography (CTCA) showing an ectatic RCA with fistulation to the coronary sinus (CS) (red arrow). Panel E: CTCA Posterior view showing the CS (blue arrow), the ectatic RCA (purple arrow), and the location of the fistula (green square). Panel F: CTCA Anterior view showing the RCA (purple arrow), the anterior descending artery (Blue arrowhead), a marginal artery (white arrowhead), and the circumflex artery (red arrowhead).

Coronary arteriovenous fistulas are rare, with an incidence of 0.002%.^1,2^ The majority of these are congenital^2^ and asymptomatic at presentation.^3^ Coronary steal can lead to several complications, including ischemia.^1,3^ The treatment approach remains controversial. The presence of symptoms, significant left-right shunt, and large fistula are the most frequent indications for percutaneous or surgical approach.^4,5^

## Data Availability

The underlying content of the research text is contained within the manuscript.
